# Toxicology

**Published:** 1850-03

**Authors:** Wm. Nofsinger

**Affiliations:** Rockville, Ind.


### ARTICLE VII.

*Toxicology*·—By Wm. Nofsinger, M.D., of Rockville, Ind.

Our Creator, has so constituted us, that we innately possess the faculty of discriminating, to a great extent, between what is noxious and innoxious—what is conducive, or injurious to health. This faculty, however is not peculiar to man. That portion of the animal creation, not endowed with reason, possesses that faculty, even to greater perfection than he. Yet his reason, is an abundant and noble compensation for whatever defect there may be in this instinct. But to a greater or less extent, with but little or no education, all animated nature distinguishes between what is beneficial or hurtful. While we are naturally attracted by the smell and appearance of wholesome food, we instinctively fly from the fetor of a decaying carcass. While the Herbivora, without any teaching, roam with delight over the beautiful meadow, and eat its wholesome grasses, they as naturally shun the *poisonous stramonium*. Then by an irresistible instinct which *our Creator* for wise and beneficent purposes, has implanted in our constitution, those substances which are salutary, naturally attract, while those of an opposite quality, naturally repel; and in consequence of the controling influence of this great principle, mankind from time immemorial, or I may say from the beginning of their race, have drawn a broad line of demarcation---on the one side having placed all those substances not hurtful to the animal system, and on the other all those substances which they supposed were hurtful, and which they denominated "*poison*."—This grand classification, no doubt, has been highly useful to man. The word "poison" has often deterred the ignorant and imprudent from meddling with substances, which in their hands, would be likely to prove only disastrous. Therefore, it is wise to label all active substances 'poison' and place them beyond the reach of such. This classification, likewise, in a very general sense may be true; yet scientifically and philosophically speaking it is subject to a very extensive modification. For instance, we shall presently see that there is not in nature any such thing as an absolute and independent poison; that nothing can be philosophically denominated poison, except in a relative sense. This declaration brings us at once to ask the question, What is a poison? What constitutes a poison? When we come to investigate the nature of a poison we can only admit that to be a poison which violates the laws of health and life. If this be a correct definition of poison then obviously the poison depends upon quantity as well as quality. It must depend upon habit, time, mode of application, and a variety of other circumstances. When we come to take these things into consideration, we will not only find poisons mere relative agents---but we will sometimes find things to be poison which have not been recognized. For instance, if a man by eating a turnip or sad biscuit produce a neuralgic pain in his stomach, and this pain be removed by the administration of one drop of Prussic Acid, the former acted as poison, while the latter proved a salutary medicine.

But before we proceed to the investigation of the nature of poisons, it may not be improper to make a few remarks in explanation of the necessity of such investigation. If the above general definition of poison be correct, then all disease, which is not the result of some mechanical cause, must be induced by some form of poison. (And indeed this is the substance of the definition of our most able Medical Lexicographers.)—And as the study of medicine naturally and necessarily embraces the causes of all pathological states of the system, the study of Toxicology, therefore, becomes imperative. If we understood the nature of those poisons which occasion all contagious, and all non-contagious epidemics, the dark clouds of ambiguity and disappointment which have ever hung over the practice, in these diseases, would be driven away. Those diseases would be disarmed of much of their terror; for their treatment, instead of being doubtful and empirical, would become certain and philosophical. In the philosophical treatment of a large majority of the disorders which afflict the human family, the two grand indications to be filled, are either to neutralize the poison, which causes the disease in question, by its appropriate antidote, or, to counteract its effect; and usually to do both. But how can we employ the proper antidote when we are ignorant of the nature of the poison? This may be, and is sometimes done, yet there is great danger, when we strike at disease in the dark, of inflicting an occasional blow upon the patient. Our ignorance of the nature of those poisons which cause our endemics, as well as epidemics, whether contagious or non-contagious, has based the treatment of these diseases upon mere semeiology. And it is true we may never detect the nature of many of these subtle poisons of those diseases, yet the long list of poisons and Alexipharmics which the light of science has already discovered to us, should encourage us, at least to make the effort to increase the catalogue. We have by the aid of Chemistry discovered antidotes for many of the mineral poisons. Arsenic, the great king, which stands at the head of mineral poisons, may be rendered powerless by its antidote, Hydrated Sesquioxide of Iron. With regard to vegetable poisons, we have not been so fortunate. For this class of poisons we have discovered but few antidotes, and even these are of uncertain character. And in relation to animal poisons we have been still more unfortunate—not having discovered the first one. It would, no doubt be desirable to know the nature of every poison, but this knowledge is not absolutely necessary in every case, to a successful application of the proper antidote. Of this fact we have an important example. There is a poison which by common consent has been denominated Malaria. This poison from time immemorial has been a scourge to many portions of the world, and has swept thousands upon thousands into their graves, yet we are still profoundly ignorant of its nature. But fortunately for mankind, after it had reigned unmolested for thousands of years, we have discovered in the various preparations of the Cinchona an antidote which, to a great extent has arrested its destructive career, and rendered it almost harmless. It may not be philosophically true to say that Cinchona is an antidote to Malaria; for we do not know whether the former neutralizes the latter or merely removes its effects.

Having premised these remarks, let us next look more closely into the nature of poisons. We have said that there is no such thing as absolute poison. What we mean by an absolute poison is that which possesses the necessary and natural power to poison under all circumstances. Now we deny that any such poison exists in nature, unless it be Hydrophobia, and analogy would seem to prove that even this is not an absolute poison. If the toxical effect of a thing depends upon the state of the system at the time of application, mode of application, habit, kind of subject, quantity, or any other circumstance, then the poison is relative and not absolute. First as to the state of the system. Many persons in the commencement of the cold stage of an ague, may, without danger of intoxication, drink a pint of dilute Alcohol. Whereas if taken at any other time it would cause them to be what is vulgarly called "dead drunk," or might as it does in some instances, destroy life.—In many spasmodic affections, a person may take more than double the amount of narcotic substances, without danger of narcosis, which he could were this condition of the system not present. Again, if a person has not had small pox, or had his system protected against this malady, the least possible amount of variolous matter, if introduced into the system, is sufficient to produce the disease: whereas if the person has had small pox or had his system protected by vaccination, no amount of the small pox virus, however great, can (as an ordinary result) generate the disease in his system. 2nd. Habit. This modifies the action of almost every substance upon the animal system. By the habitual use of many substances the system may be enabled to bear with impunity an amount which would otherwise destroy it. We are furnished with abundant examples of this fact in the wide spread use of tobacco, opium, and alcohol; as well as our daily experience in the action of almost every remedy employed in the treatment of disease. 3d. Mode of application. This would comprise the difference in the action of substances applied to different parts of the economy. For instance, Carbonic Acid may be taken into the stomach in safety, but if inhaled into the lungs would produce asphyxia and death. Sulphureted Hydrogen Gas may be present in the bowels without inconvenience, whereas if introduced into the lungs it would prove even more deleterious than Carbonic Acid. Many substances may be safely applied to the skin, which cannot at all, in quantity, be introduced into the stomach. Yet there are some substances, which may be taken into the stomach with impunity, which if endermically applied, would in many instances cause death. Such is true in relation to the venom of the Rattle Snake, Viper, &c. 4th. *Quantity*. By this is meant the amount of any given substance, necessary to produce a poisonous effect. There has never yet been a substance analyzed, which has not been administered in certain quantities, with entire safety. Those substances which have been considered as the most active poisons have been given again and again in certain minute quantities, without the least deleterious consequence. We will take the liberty to say that, it is entirely reasonable to suppose, that there is not in nature, including all the great kingdoms of matter, a substance which may not be given in some quantity, without any poisonous result; unless it be some substance, capable of rapid reproduction, and development in the animal system, as is said to be true with regard to some species of animalculæ, and more especially some species of microscopic plants, belonging to the class cryptogamia. It is said that species of plants belonging to this class of vegetation, will multiply billions of times in 24 hours. If this be true, we may very readily perceive how the system may be poisoned by even one seed, at least by a few *Semina Morborum*, which could not be discovered by a microscope magnifying hundreds of times! This will give a solution to the fact that so small an amount of virus will produce variola. Of course we cannot demonstrate that the virus of small pox is either animacular or cryptogamous, yet its *modus operandi*, would rather favor the hypothesis.—5th. The kind of subject. By this we mean, not only the species of animal we experiment upon, but also allude to individual peculiarities in the same species. As for instance, we find, that in relation to man, there are some substances, which experience proves most salutary food to some individuals, while they are invariably noxious to others. The exhalations from the Rhus Toxicodendron poison some, while to others they are wholly innoxious. This idiosyncrasy, or peculiar constitution is exemplified, every day, in the practice of medicine, and confirms the homely old adage that "what is one man's meat is another's poison." But when we look abroad into the animal creation, we find a still further confirmation of the peculiarities of the animal system, in relation to toxical agents. For example, while the mountain Ivy or Laurel, (Kalmia Latafolia) is a healthy food to the deer, goat, partridge, and pheasant, it is a deadly bane to sheep and other animals. While Aloes are a safe and salutary cathartic for man, they are death to dogs and wolves. And while the ox can eat with impunity the Water Hemlock, (Phellandrium Aquaticum) it kills the horse. Many similar facts could be adduced, but those already referred to are deemed sufficient to establish beyond controversy, the truth that all poisonous or toxical agents are dependent upon some condition or circumstance; and the deduction, therefore, is irresistible that such agents are relative, and not absolute ;—and that such a thing as an absolute and independent poison is a mere ideal personification,—inconsistent with the laws of nature, and can only exist in the wild regions of fancy. A Poison, perhaps, may be defined to be a thing which cannot exist according to law, but is dependent for its existence upon violation of law!

Having made these remarks in regard to the general nature of poisons, we will now introduce Dr. Christison's classification which is generally adopted. It is as follows, viz:

1st. Irritant poisons, or those which produce irritation and inflammation, as the mineral Acids, Oxalic Acid, Arsenic, Mercury, Copper, Zinc, Lead, Baryta, and Cantharides."

2d. "Narcotic poisons, or those which produce stupor, delirium, and other affections of the brain and nervous system, as, Opium, Hydrocyanic Acid, and poisonous Gases."

3d. "Narcotico-acrid, poisons, which produce sometimes irritation, sometimes narcotism, sometimes both together,—these are all derived from the vegetable kingdom, as Strychnia, Nux Vomica, and poisonous Fungi."

Dr. Hoblyn remarks that poisonous "Substances are those which derange the vital functions and produce death by an action not mechanical." If this definition be correct, and if poisons are mere relative agents, we think we may introduce a class or kind of Toxical agents, which have not heretofore been recognized as such. These agents consist in Atmospheric changes, not only in its inherent and healthy constitution, but in reference to its Electric, Thermometric, and Hygrometric states. That such changes act deleteriously upon the animal system—that they violate the laws of health and life, is as clear and as susceptible of demonstration as that Ratsbane in certain quantities will destroy life! Indeed, no one can deny that these changes are constantly occuring, and that they derange the vital functions by an action not mechanical, thus producing a vast amount of disease and death, and may, therefore, be strictly denominated poisons. But before we enter into a minute examination of this description of poisons, we will first take a cursory view of each class of the above named poisons, in their regular order. First then in order are irritant poisons, or those which produce irritation and inflammation. How do these agents poison? Manifestly by a direct action upon the solids and fluids of the animal system. These are directly decomposed. Take for example Sulphuric Acid. When this substance in a concentrated state is applied to the animal tissues, there is mutual decomposition.—This result may be known *a priori*, when we consider the composition of the tissues, and that of the Acid. The ultimate elements of all animal tissues, are Oxygen, Nitrogen, Hydrogen and Carbon. Those of the Acid are Sulphur and Oxygen. Now why this decomposition when the Acid and tissue are brought in contact? Simply because you bring into play more powerful affinities than those by which the composition of the tissue and Acid are maintained. Owing to the electro-positive and electro-negative states of these various elements, the Oxygen of the Sulphur has a stronger affinity for either the Nitrogen, Hydrogen or Carbon, than it has for the Sulphur, therefore it leaves the Sulphur and unites with these several elements, but chiefly with Hydrogen and Carbon, forming with the first water, and with the latter Carbonic Acid. But owing to the greater affinity between Nitrogen and Hydrogen, than between Nitrogen and Oxygen, the Nitrogen unites with the Hydrogen, forming Ammonia. This then is the rationale, which must explain, at least all highly irritant poisons. And if this be true, then intense heat should be classed as an irritant poison, because it produces precisely the same effect upon the animal tissues just above detailed Heat is the most common, and next to electricity, the most powerful agent in effecting chemical changes and decompositions. Heat has the power, when applied to the tissues, of changing the electro-negative, and electro-positive conditions of the elements of the tissues, and hence their decomposition.

But Narcotics, the second class of poisons are much more obscure in their actions. Here you have no lesion of the animal tissue. How then do they exert their poisonous effect? All admit this description of poisons powerfully affects the brain and nervous system;—and whether they previously act upon the fluids of the system is not known. It is a custom when we cannot demonstrate a subject, to resort to analogy to explain whatever phenomena attract our attention. The manner in which Narcotics produce their poisonous effects has ever been a subject of the deepest interest. The laws of vitality are not well understood. Yet, notwithstanding their obscurity, we are sufficiently satisfied that these laws greatly modify the action of all physical laws upon the animal economy.—Therefore, we cannot logically suppose, that the same physiical results take place in the animal system, which we see occurring out of it. But it is equally as logical to presume a similarity of cause where we observe similar phenomena.—We have often observed phenomena developed by the action of electricty, upon the animal system, which frequently take place spontaneously, or rather from the influence of some disease of the brain and nervous system, or when some highly exciting application has been made to the system. Hence it has been inferred that the electric and nervous fluids are similar. And the brain and nervous system have been, not inaptly, compared to a galvanic battery, whose office it was to secrete and collect the necessary fluid for the innervation of the system! To strengthen this view of the subject we may remark that the phenomena of sneezing, coughing, and indeed all convulsive actions are similar to the action occasioned by electricity----and in conclusion upon this topic, we simply remark, that it is not improbable, Narcotic poisons, destroy life in the same manner that electricity does---that is, by suspending the action of the brain and nervous system, or producing a derangement in these organs incompatible with the functions of life. Nothing need be said in relation to Acro-Narcotics, or the third class of poisons, any farther than to remark, that they exhibit properties belonging to the two classes already detailed. The explanations of the first and second classes embrace that of the third. Therefore, we will proceed at once, to the consideration of those Toxical influences or agents, which consist in Atmospheric changes----including alterations in its electric, thermometric and hygrometric states.

1st., in relation to changes in the natural and healthy composition of the atmosphere. A healthy atmosphere, according to the analysis of the best chemists consists, of a mixture of four parts of Nitrogen to one of Oxygen, and in every two thousand parts of such mixture one of Carbonic Acid, and in addition to these, it always contains an amount of water, heat, and electricity. These last three ingredients, no doubt may be somewhat variable in quantity, without materially affecting its healthy constitution. There is manifestly a conservative principle in nature, which constantly operates to maintain a healthy proportion of these various ingredients. Yet this principle is often opposed by disturbing causes; and no argument need be introduced here to show the infinite amount of disease, which is constantly occurring in consequence of such disturbance. The essential constituents of the atmosphere being Nitrogen and Oxygen, experiment has shown that the slightest variation in their proportions, is immediately fatal to animal life. Carbonic Acid of the atmosphere may possibly be diminished, but evidently cannot be much increased without disease and death. Vats of distilleries, cellars, wells, and other low places, in which this gas accumulates on account of its greater gravity, have often given us fatal proof of the deleterious effects of an increase of the proportion of this gas.

2nd. *Electric state of the Atmosphere.* That changes in the electric state of the atmosphere, give rise to disease is a fact too well established to require special facts as proof. The effect of a diminutive of the normal quality of electricity in the air is to depress the mind and feelings, and to lower the tone, and relax the animal system generally—causing a predominance of the excrementitious secretions, thereby giving rise to catarrhs, fluxes, diarrhœas, &c. But on the other hand where the electricity is increased, the animal system is too highly stimulated---the recrementitious secretions predominate, and an inflammatory diathesis of the system is thus induced, which lays the foundation of a great variety of inflammatory diseases.

3d. *Thermometric state of the Air.* Very little observation is necessary to establish the fact that changes in the temperature of the atmosphere, if they take place very suddenly, are deleterious to health. Yet there are numerous instances to prove that the animal system evidently possesses the vital power of regulating its own proportion of heat; this is obvious from the fact that the temperature of the human system is no higher under the burning sun of the tropics, than amid the icebergs of the north. We have not had much opportunity of investigating the subjects of Zoology and Ornithology in reference to the subject of temperature, but so far as we have, the paradoxical fact presents itself, that the lower animals and the birds of the North have a higher temperature, than those of the South. This would seem also a natural result, of the necessity of greater energy in the calorific function in the North, than in the South.

*Hygrometric State of the Air.*—This, no doubt, may be variable without injury to the animal system, yet it is not reasonable to suppose that the normal quantity of water in the air could be greatly increased or diminished without the most deleterious consequences. A large quantity of water in the atmosphere is usually accompanied with a *deficiency* in the amount of electricity, and would seem to produce, or assist the latter in producing those diseases already named. But an anhydrous air is almost irrespirable, and produces the most fatal effects upon the animal system. This, no doubt, is the principal abnormal state of the air of those pestilential winds which arise from the parched deserts of Africa and Arabia. These winds are known in Africa as the Samiel, or Simoom. They blow westwardly, crossing the Mediterranean Sea, and are lost upon the European Continent. In Italy they are known as the Sirocco. But an important fact should here be obsereved,---these winds are much less poisonous and destructive to human health after crossing the Mediterranean, than they are upon their native shore. How do we account for this fact? Manifestly by supposing, that the healthy hygrometric state of the air is restored by crossing the sea. Although these winds are greatly improved before they reach I taly, they are still unhealthy. But in what their remaining unhealthiness consist, we have not now time to conjecture.

These hastily written general views, which we have submitted on the subject of Toxicology, display to us a field illimitable, the exploration of which is scarcely commenced.

